# Intermittent Fasting as a Potential Therapeutic Instrument for Major Depression Disorder: A Systematic Review of Clinical and Preclinical Studies

**DOI:** 10.3390/ijms242115551

**Published:** 2023-10-25

**Authors:** Laís Murta, Daniela Seixas, Luana Harada, Rodolfo Furlan Damiano, Marcus Zanetti

**Affiliations:** 1 Hospital Sírio-Libanês, Sao Paulo 01308-050, Brazil; luanaharada@hotmail.com (L.H.); zanetti.mv@gmail.com (M.Z.); 2 Faculdade de Medicina, Universidade de São Paulo, Sao Paulo 01246-903, Brazil; dseixas@yahoo.com (D.S.); damianorf@usp.br (R.F.D.)

**Keywords:** intermittent fasting, restricted feeding, depression, disorder, major depressive, major depressive disorder, mood, mood disorder

## Abstract

Recent studies have reported positive effects of Intermittent Fasting (IF) on metabolic parameters, cognition, and mood. However, regarding depressive symptoms, the effect of IF is not clear. The purpose of this review was to assess the available evidence on IF interventions for depression in both clinical and preclinical studies. Of the 23 included studies, 15 were performed on humans and 8 on animal models. The studies on rodents suggested that IF acts as a circadian regulator, improving neurotransmitter availability and increasing the levels of neurotrophic factors in the brain. However, the investigations on humans mainly evaluated healthy volunteers and showed a great heterogeneity regarding both the IF regimen studied and the observed effects on mood. Most available clinical trials have specific limitations, such as small sample sizes and uncontrolled designs. A comprehensive systematic review was conducted on five databases, PubMed, Cochrane, the Central Register of Controlled Trials, Web of Science databases, BVS and Scopus, identifying 23 relevant studies up to 6 October 2022. IF has potentially relevant physiological effects for the treatment of mood disorders, but better designed studies and controlled evaluations are needed to evaluate its efficiency in the treatment of major depression.

## 1. Introduction

Major Depressive Disorder (MDD) is the most disabling mental disorder in the world and is the second most prevalent after anxiety disorder [1,2]. Until 2020, it was estimated that 193 million people were affected by Major Depressive Disorder (MDD) [2]. In the wake of the COVID-19 pandemic, this figure surged by 28%, reaching 246 million people, according to data released by the World Health Organization in 2022 [3]. Although psychopharmaceuticals and psychotherapeutic interventions are the main treatment modalities for depression, these treatments often do not produce the expected results, with approximately 50% of patients with MDD presenting a condition resistant to the conventional treatments [4,5], and the risk for relapse increases with each subsequent episode (70% and 90% after a second and third episode, respectively) [6]. Therefore, this high prevalence, as well as the high rates of recurrence and resistance to treatment of MDD, entailing enormous costs for society, are motivators for the search for new therapeutic interventions. In this sense, there is a growing interest in reducing the risk of depression in the population through lifestyle interventions, particularly nutritional strategies [7,8].

Evidence for the importance of lifestyle habits and environmental factors means they are increasingly recognized as central to the pathophysiology of MDD, since the genetic heritability of MDD is limited to approximately 30–40% of cases [9,10]. In addition, studies on the genetics of MDD have found an interesting overlap with genetic risk variants for metabolic syndrome [11,12]. Both in MDD and in other mental illnesses, including neurodegenerative disorders, chronic systemic inflammation, high oxidative stress, insulin resistance, and changes in the microbiota–gut–brain axis (GMBA) have been observed as potentially harmful, etiological, and/or aggravating factors, contributing to so-called neuroprogression [13,14,15,16].

Different nutritional strategies have been proposed over the past 20 years, each with specific characteristics, enriched or devoid of certain components [17]. The Mediterranean diet and its derivations are the most effective therapeutic options that have been evaluated until this moment, such as those used in the PREDIMED [18], SMILES [19], HELFIMED [20], and AMMEND [21] studies. Furthermore, studies evaluating carbohydrate-restricted diets and the ketogenic diet have reported potential benefits in terms of improving and maintaining mood in individuals with mood disorders, as pointed out in a recent systematic review that included 12 studies, including 9 case series, 2 cohorts, and an observational study [22].

Over the last two decades, different studies have suggested that intermittent fasting (IF) may be a nutritional intervention potentially beneficial to physical and mental health [23]. IF can be characterized as periods of voluntary abstinence from food and beverage consumption, an ancestral practice followed, in very varied ways, by populations around the world [24]. The main types of intermittent fasting are shown in Table 1. Evidence of different natures (studies on animal models and on human populations) document a series of the potentially beneficial physiological effects of IF, including the induction of cell maintenance and repair mechanisms, an improvement in insulin response, the suppression of inflammation, and an increase in the production of neurotrophins, in addition to beneficially modulating the GMBA [23,25,26].

Despite the large number of preclinical and clinical studies evaluating the metabolic effects of IF, there are only two systematic reviews to date that have evaluated the impact of different IF practices on mood [27,28]. The studies evaluated the effect of IF on anxiety and depression, with potentially positive effects on depressive symptoms, but none of them evaluated MDD specifically, nor the underlying mechanisms of IF on mood. Although the coexistence between MDD and anxiety disorders is common—about 45% of individuals with MDD have a history of one or two episodes of anxiety disorder throughout their lives—it is important to emphasize that the pathophysiological mechanisms and clinical manifestations of both conditions are different, factors that influence the direction of therapeutic conduct [29]. Considering these differences, the aim of this systematic review is to evaluate the available evidence and its quality on the potential effects of IF in the context of MDD treatment, in addition to investigating the physiological mechanisms underlying the effect of IF on mood, as well as to evaluate, among the existing IF protocols, if there are any with greater evidence of benefits in terms of improving depressive symptoms.

**Table 1:** Main types of intermittent fasting.

Fasting Paradigms	Characteristics
Alternate Day Fasting (ADF)	Ad libitum feeding cycles for 24 h alternated with 24 h of total fasting (no caloric intake). Modified alternate day fasting (25% of caloric needs maintained; consumption of approximately 500 kcal on “fasting” days) is the predominant type of IF in the literature [30,31]
Two Days Fasting	Two consecutive or non-consecutive “fast” days, followed by five days of ad libitum food (a 2-day fast is popularly known as a 5:2 fast) [32]. Fasting days can be either full fasting or keeping to 25% of caloric needs. Also, they can be consecutive or non-consecutive [31,32].
Time-Restricted Feeding (TRF)	This type of fast limits food intake to a daily eating window of 4–10 h, promoting a fasting period of 14–18 h. This intervention reduces the opportunity to eat, tending to reduce food intake. However, it does not necessarily imply caloric restriction [33]. An early restriction of the feeding window involves shifting the feeding window to an earlier part of the day, from morning to mid- or late afternoon, aligning feeding periods with the body’s circadian rhythm [32].
Religious Fasting	Fasting is an ancestral practice, present in different religions. Although religious fasts have spiritual purposes, they can also impact physical and mental health in different ways [33]. The most extensively studied religious fast for health effects is the Ramadan fast (JR). Ramadan is one of the five pillars of Islam and occurs during the ninth month of the Islamic lunar calendar, in which healthy adult Muslims abstain from the consumption of food and fluids from dawn (el fajr) until sunset, for approximately 30 days. The duration of the daytime fast varies and is significantly impacted by location and season. In general, the typical duration of the fasting period is 10 h, but it can exceed 18 h [34].

## 2. Methods

We performed a systematic review following a pre-registered protocol on PROSPERO (CRD42022343444), adhering to the PRISMA reporting guidelines.

### 2.1. Search Strategy

The search was carried out in PubMed, Cochrane, the Central Register of Controlled Trials, Web of Science databases, BVS and Scopus for peer-reviewed studies published from the database inception until 5 October 2022. The search terms included: (“Intermittent fasting” OR “restricted feeding”) AND (depression OR depressions OR “disorder, major depressive” OR “disorders, major depressive” OR “major depressive disorder” OR “major depressive disorders” OR mood OR “mood disorder” OR “mood disorders” OR “disorder, mood” OR “disorders, mood” OR “bipolar depression” OR “bipolar disorder” OR “bipolar disorders”).

Two investigators (LDMN and MVZ) independently screened the titles and abstracts of all the articles retrieved from the search and reviewed the full texts of the potentially eligible studies. Inter-rater agreement was high (94.8%) and remaining disagreements were resolved by discussion, consensus and, when necessary, resolution by the senior author (MVZ).

### 2.2. Study Selection

We included studies that:

1. Included clinical intervention studies on human populations of any age group in which a diagnosis of depression or the presence of depressive symptoms had been assessed using standardized instruments.
2. Prospective or retrospective cohort population studies evaluating the association between feeding windows and/or mealtimes and affective symptoms, and their possible underlying biological markers.
3. Preclinical studies evaluating the physiological effect of intermittent fasting strategies in animal models of depression or behavioral activation.

### 2.3. Data Extraction

**Figure 1:**
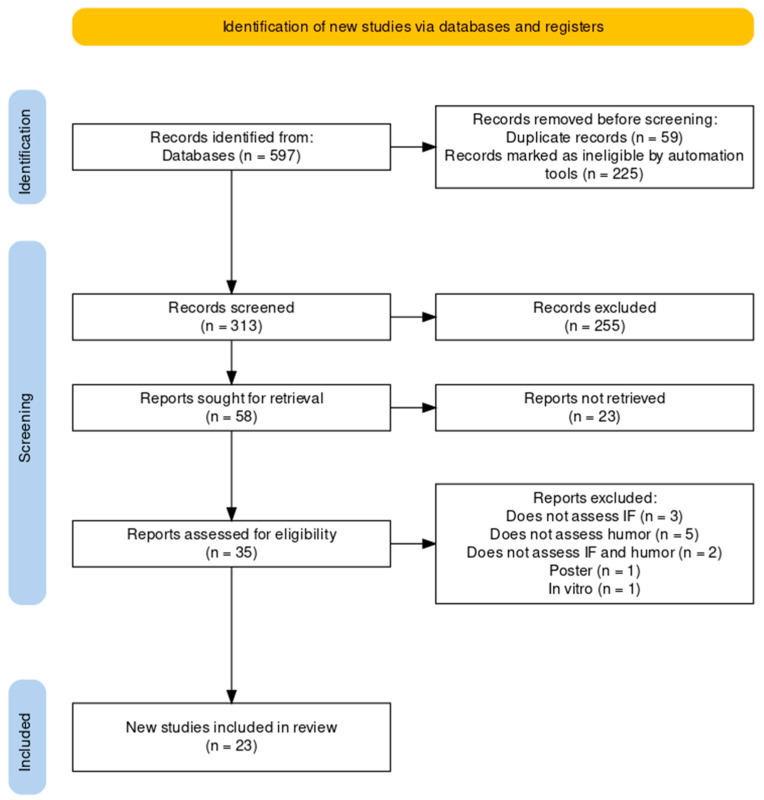
Preferred Reporting Items for Systematic Reviews and Meta-Analyses (PRISMA) flow chart depicting the studies included in the meta-analysis. The figure was created with and based on Haddaway et al. (2022) [35].

The database search resulted in 597 records. No additional studies were identified in the clinical trial registers or in the references of the included studies. After deleting duplicates, 342 records remained. After the initial evaluation based on the titles and abstracts of the studies, we identified 35 relevant records. After reading the articles in their entirety, 12 of the 35 studies were excluded. Thus, 23 studies met the inclusion criteria and were included in our systematic review. The search results flow chart is shown in Figure 1.

## 3. Results

Of the 23 studies included, 8 were on animal models (Table 2) and 15 were on humans (Table 3). From the human studies, we had: eight randomized controlled clinical studies; one controlled, non-randomized clinical trial; five case series; and one retrospective cohort study.

*a*. *Description of animal models studies included*

The eight animal model studies included evaluated the effects of IF on insulin resistance and body composition [36,37], serotonergic transmission [38], neurotrophic factors [36,38], circadian regulation [39,40,41,42], and the impact on the intestinal microbiota [43]. Different models of mood and depression were used in these studies, including the forced swim test (FST), open field test (OFT), elevated plus maze test (EPMT), splash test, sucrose preference, novel object recognition test, time-to-exhaustion test, Morris Water Maze (MWM), spontaneous locomotor activity (SLA), and feeding and hydration behavior. In this systematic review, most studies on animal models found (four in total) evaluated the effect of IF on circadian regulation. A description of the animal studies is presented in Table 2.

*b*. *Description of humans studies included*

This systematic review included 15 human studies (the details of which are summarized in Table 3). These evaluated very diverse IF paradigms, namely: RF [44,45,46,47,48] 24 h fasting [49,50], TRF for 8 h a day [50,51,52,53], fasting for two days, consecutive or not [54,55,56], with or without physical exercise [54], and probiotic supplementation [56]. Of the 15 studies reviewed, only 8 were randomized, placebo-controlled intervention studies [48,50,51,54,55,56,57] and 1 of them was a retrospective cohort study [58].

The studies also varied in terms of caloric intake recommendations, the presence of a control group, intervention duration, and the health status of the evaluated individuals. Only one randomized controlled trial was conducted on patients diagnosed with MDD [48], and all the others were carried out with psychiatrically healthy individuals. Six studies associated caloric restriction with IF [49,50,54,55,56,57], while the others allowed ad libitum feeding during the feeding period. None of the clinical studies analyzed the nutritional quality of the diet.

**Table 2:** Studies evaluating intermittent fasting or restriction of the eating window in animal models of depression.

Study	Animal	Behavior Models	Experimental Design	Additional Measures	Main Results
[36]	Male mice	Run to exhaustion test	Six groups: ADF DF, CR, HFD ad libitum, standardized rodent chow (control) for 8 weeks.	Glucose tolerance, OGTT test, MRI	Except for the HFD group, all other groups showed improvement in body composition, physical disposition (longer running time until exhaustion), and insulin sensitivity (reduced fasting glucose and HOMA-IR).
[37]	Healthy and DM2 male rats	FST and EPMT	Four groups: Group I (healthy rats) ad libitum diet (controls); Group II (healthy rats) 18 h daily fasting (14:00–8:00 h) for 3 months; Group III (DM2 induction) with a high-fat diet for 4 weeks followed by a single intraperitoneal injection of streptozotocin; and Group IV DM2 rats exposed to IF for 3 months.	BDNF, neurotrophin 3 (NT3), serotonin, dopamine, and glutamate levels in the hippocampus	It was observed that IF reduced anxiety and depression and increased neurogenesis markers BDNF and NT3 in the hippocampus, both in the control group and in DM2 rats; IF improved metabolic dysfunction and reduced corticosterone levels in rats with DM2.
[38]	Male mice	FST and OFT	Five groups: without fasting (negative control) versus 3 h of fasting versus 9 h of fasting versus 18 h of fasting, with the use of imipramine.	Brain levels of the transcription factor CREB and its phosphorylated form (CREB-p); possible involvement of 5-HT2 receptors was examined using the HT2A/2C receptor agonist DOI	Fasting for 9 h, but not for 3 h or 18 h, significantly reduced the immobility time without changing the locomotor activity in the co-administration of 9 h of fasting with imipramine produced an additive antidepressant effect, in addition to an increase in the p-CREB/CREB ratio; these effects were partially reversed using DOI.
[39]	Male mice	MWM	Three groups: ADF, CR or ad libitum (control), for three months.	MWM (for assessment of learning and spatial memory); neuroblast count (doublecortin-positive cells); and Klotho gene expression in the hippocampal dentate gyrus of mice	It was observed that ADF was superior to CR in increasing hippocampal neurogenesis and inducing Klotho gene expression and was also superior to CR in improving spatial memory.
[40]	Male rats	SLA, drinking and eating behavior	Three groups: rats fed ad libtum diet or 2 h/d during light (TRF) and with or without colchicine induced VMH disruption.	Rhythmicity of variations in temperature, food, water consumption, corticosterone level, and cellular expression of c-Fos in the hypothalamus during the light/dark phase	There was a reduction in SLA, an increase in food consumption, water consumption, body weight, and insulin levels in the group with VMH disruption by colchicine; TRF induced the VMH rhythm (assessed by increased c-Fos activity) and reduced food intake and weight in the group with VMH disruption by colchicine; it was observed that the VMH acts as an amplifier of stimuli coming from the SCN.
[41]	Male rats	SLA	Two groups: rats with hereditary microphthalmia and normal rats (control), submitted to TRF (6 h during the light period).	Pineal serotonin N-acetyltransferase	Rats with hereditary microphthalmia maintained the ability to alternate and synchronize circadian phases when subjected to FRT, even with loss of the optic nerve (and light-induced circadian clock desynchronization); a peak of pineal enzyme activity was observed during the feeding period in the blind rats.
[43]	Male rats	OFT, sucrose preference, novel object recognition test	Two groups: shiftwork (wakefulness and forced activity through rotating drums, to which the rats were submitted for 8 h) and ad libitum access to food (W-AL) and shift-work and TRF during the active phase (W-TRF).	Body weight, food consumption, cell markers of astrocytes (GFAP) and microglia (IBA-1) in different brain structures	It was observed that TRF prevented the occurrence of depressive and anxious behaviors in addition to reducing neuroinflammation markers (induced by the shift-work experimental condition).
[43]	Male rats	OFT, EPMT, Splash Test	Four groups: sedentary controls (SC), trained controls (TC), sedentary + IF (SIF), and trained + IF (TIF), evaluated for four weeks; intervention: 3 p.m. TRF (5 p.m.–8 a.m.); controls: ad libitum diet for 9 h (8 h–17 h), for 28 days.	Adiposity index, analysis of fecal microbiota, quantification of organic acids in the intestine (colon) and feces, and dosage of IL-1 β in the hippocampus	There was a reduction in anxious behavior and an increase in IL-1β markers in the TIF group. IF and aerobic exercises, associated or not, modulated parameters related to GMBA, such as an improvement in anxiety and depression and quantification of organic acids in the intestine and feces, but they did not act synergistically.

ADF, Alternate Day Fasting; BDNF, Brain-derived Neurotrophic Factor; CR, Caloric Restriction; CREB, cAMP response element-binding protein; DF, Daniel Fasting; DM2, Type 2 Diabetes; DOI, (±)-1-(2, 5-dimethoxy-4-iodofenil)-2-aminopropano hydrochloride; EPMT, Elevated Plus Maze Test.; FST, Forced Swin Test; GMBA, Gut–Microbiota–Brain Axis; HFD, High-Fat Diet; IF, Intermittent Fasting; MRI, Magnetic Resonance Imaging; MWM, Morris Water Maze; NES, Night Eating Syndrome; OFT, Open Field Test; OGTT, Oral glucose tolerance tests; SCN, Supraquiasmatic Nuclei; SLA, Spontaneus Locomotor Activity; SSRI, Serotonin Selective Reuptake Inhibitors; TRF, Time-Restricted Feeding; and VMH, Ventromedial Nucleus.

**Table 3:** Studies on the effect of intermittent fasting or time restricted feeding on mood symptoms in humans.

Study	Design	Segment	Participants	Intervention or IF Paradigm	Assessment Tools	Main Results
[44]	CS	28 days	10 healthy men (age: 20–28 years)	RF	Oral temperature (as a circadian assessment measure); CFF; VAS; Choice Reaction Time, administered 6 times a day: at 09:00, 11:00, 13:00, 16:00, 20:00, and 23:00 on the 6th, 15th, and 28th days	There was a reduction in oral temperature during the day and an increase at night; there was a worsening of the subjective state of alertness and mood (VAS) during the RF.
[45]	CS	7 weeks	10 healthy men (age: 22.06 ± 1.98 years)	RF	VAL, POMS, one week before the RF, in the 3rd week, 4th week, and 2 months after	No differences in neuromuscular efficiency and resting twitch potential; higher depression, fatigue, and anxiety (POMS) scores were observed during RF.
[46]	CS	6 weeks	34 healthy subjects (19 men, age 24.8 ± 1.0 years; 15 women, age: 25.5 ± 1.20 years)	RF	FSS, ESS, BDI-II, HADS	An improvement in morning fatigue was observed in both genders; improvement in mood and depressive symptoms in men (HADS and BDI-II); and significant reduction in muscle mass and body water in women.
[47]	CS	1 week of usual diet followed by 4 weeks of intervention (RF) and reassessment 1 month after the end of the intervention	34 healthy subjects (19 men, age 24.8 ± 1.0; 15 women: age 25.5 ± 1.2 years)	RF	BDI-II, HADS-A/D, QoL, FSS, ESS scales; Cortisol, BDNF, IL-8, MMP-9, IGF-1, and myoglobin levels in the blood; assessments were performed one week before, during, and up to 1 month after the RF	There was a significant reduction in BDNF levels during and up to 1 week after RF, which correlated positively with the HADS-Dscore; only in women was there a significant reduction in cortisol after 1 week of RF that correlated positively with the score on the BDI-II.
[48]	RCT	4 weeks	100 men with MDD: Intervention group (n = 50, age: 43.38 ± 10.06 years), control group (n = 50, age: 46.54 ± 8.35)	RF	PHQ-9, IPAQ-SF, plasma lipid levels, and blood pressure	There was no difference in depressive symptoms (PHQ-9); there was a reduction in weight, BMI, and body fat.
[49]	CT	8 weeks of intervention and reassessment 4 months after the end of the intervention (6 months of follow-up)	36 healthy subjects (33 included in the final data); intervention group: n = 22 (women: 15, men: 7; age: 42.45 ± 10.82 years); control group: n = 14 (women: 7, men: 7; age: 41.36 ± 12.63)	Intervention: 24 h fasting once a week, with abstinence from solid food and maximum caloric intake of 300 kcal; control: advice on healthy diet	Insulin, glucose, HOMA, BDNF, HbA1C, LDL-C, HDL-C, triglycerides), coagulation markers, liver enzymes, IGF-1, BDNF, blood pressure, waist circumference, BMI, % fat; WHO-5, HADS, POMS, FS, and VAS	There was an improvement in general symptoms of mood and depression (HADS)—as well as in insulin response—over the 8 weeks of intervention in both groups, with no significant difference between them; no differences were observed in BDNF levels; the IF group only showed a greater reduction in BF and alkaline phosphatase compared to the controls.
[50]	RCT	8 weeks	46 healthy overweight or obese women (age 50 ± 9 years, BMI 32.9 ± 4.4 kg/m^2^	24 h IF every other day (3×/week) associated with CR (70% reduction in energy requirement) compared to CR without IF (control group)	TFEQ, DASS, PSQI, AQoL, and SF-36	Weight reduction was observed in the IF + CR group; no differences were observed between groups regarding perceived food consumption (TFEQ), mood (DASS), AQoL, sleep quality (PSQI), and cognition (SF-36).
[51]	RCT	5 days	11 sedentary men (Age 38 ± 5 years; BMI: 32.1 ± 2.1 kg/m^2^)	5 days of an isoenergetic diet, with 3 meals a day between 10 a.m. and 5 p.m. (TRF; 8 h eating window) or between 7 a.m. and 9 p.m. (diet with no restriction on eating window)	PANAS and VAS	No differences in mood were observed; an increase in fatigue was observed in both conditions (PANAS and VAS).
[52]	CS	2 weeks of usual diet followed by 4 weeks of intervention	19 participants with DM2 (9 men, age 48.7 ± 10 years; 10 women, 51.6 ± 8 years)	TRF (9 h ad libitum feeding window)	DASS, AQol, PSQI, CBB, and CIA	No difference in measures of depression, anxiety, and perceived stress (DASS and AQol); there was an improvement in executive functions, specifically in the Groton Maze Learning Task.
[53]	RCT	14 weeks	90 obese subjects (age 43 ± 1 years; BMI, 39.6 ± 6.7 kg/m^2^); intervention with 35 women and 10 men versus control with 37 women and 8 men	TRF (8 h/day, from 7 a.m. to 3 p.m.); control: 12 h feeding window (7 a.m.–7 p.m.)	BPAQ, POMS-SF, PHQ-9, MCTQ, PSQI, and 5-point Likert scale; fasting blood pressure, glucose levels, insulin, insulin resistance (HOMA-IR), β-cell HOMA, hemoglobin A1c level, and plasma lipid levels; 3 days of food recall	There was an improvement in fatigue and depressive symptoms in the intervention group (POMS-SF); early TRF was more efficient in weight loss (with an additional loss of 2.3 kg).
[54]	RCT	12 weeks	Healthy men: intervention group (n = 16, age: 59.7 ± 6.6 years; BMI = 26.7 ± 1.8) versus control (n = 15 (Age = 59.7 ± 6.2 years; BMI = 26.5 ± 2.7)	24 h fasting (on Mondays and Thursdays) associated with restriction of 300 to 500 kcal/day in relation to the participants’ baseline energy intake	POMS, BDI-II, GDS-15, food recall, and fasting record at the beginning of the intervention and at weeks 06 and 12	A reduction in levels of tension, irritability, and confusion, and subjective improvement in mood (POMS) were observed; there was no difference in scores on the BDI-II and GDS-15 scales; weight, BMI, and fat percentage were reduced by 3.8%, 3.7%, and 5.7%, respectively.
[55]	CT	8 weeks	36 individuals with multiple sclerosis divided into 3 groups: control (n = 12; men: 3, women: 9, mean age: 33.3 ± 7); daily CR (n = 12; men: 2, women: 10, mean age: 40.5 ± 5.4); and intermittent CR (n = 12; men: 2, women: 10, mean age: 38.5 ± 7.4)	Control diet (100% of caloric requirement 7 days/week), daily CR (78% of caloric requirement, 7 days/week), and intermittent CR (“5:2” style diet (25% of daily caloric requirement for 2 consecutive days per week; 100% of daily caloric needs 5 days a week)	FAMS and PSQI, at baseline and during the 4th and 8th week	Daily and intermittent CR were associated with better emotional well-being and lower rates of depressive symptoms (FAMS); There was also a marginal benefit in weight loss.
[56]	RCT	12 weeks	26 pre-diabetic individuals; intervention: 6 men, 9 women (age: 52.9 ± 8.7 years); placebo: 2 males, 9 females, (age: 54.1 ± 6.4 years)	PROFAST: IF 2 days/week (non-consecutive) associated with a daily reduction of 600–650 calories and increased use of the probiotic *Lacticaseibacillus rhamnosus* HN001 (n = 15) or placebo (n = 11)	PHQ-9, GAD, and EDQ	Improvement in depressive (PHQ-9) but not anxiety (GAD) symptoms was observed in the total sample (both groups combined) over time.
[57]	RCT	12 weeks	34 young adults: intervention (n = 17; 9 men, 8 women; age: 24.7 ± 4.8 years), control (n = 17; 8 men, 9 women; age: 23.2 ± 3.9); average BMI: 27.0 kg/m^2^, average age: 23.9 years	Intervention: fasting for two consecutive days per week (5:2) with CR of 80% of energy requirement associated with resistance exercises; control: CR of 20% during the entire intervention period associated with resistance exercises	Diet adherence (self-reported), post-intervention intention survey assessing mood, hunger, and satiety levels; level of plasma lipids, glucose, insulin, CRP, and HOMA-IR	There was a positive correlation between mood and adherence in both groups; improvement in the feeling of hunger and bad mood on non-fasting days; high rate of adherence to diet (~80%) and low levels of hunger in both groups; reduction in LDL-c and HDL-c in both groups. There was no difference in markers of glycemic regulation.
[58]	Retrospective cohort	No follow-up	1572 adults divided into 2 groups: TRF < 8 h (n = 120; men: 47 ± 39.2; women: 73 ± 60.8, age: 55 ± 14.8) vs. > 8 h (n = 1452; men: 613 ± 42.2, mean age: 45.8 ± 17.1; women: 839 ± 57.8; mean age: 45.8 ± 17.1)	TRF	Food frequency questionnaires; PSQI; PSS; and CES-D-10	There was no significant difference in stress levels (PSS), sleep quality (PSQI), and depressive symptoms (CES-D-10) between groups.

AQoL, Assessment of Quality of Life; BDI-II, Beck Depression Inventory-II; BF, Body Fat; BMI, Body Mass Index; CBB, Cogstate Brief Battery; CES-D-10, Short Form for the Assessment of Depression from the Center for Epidemiological Studies; CFF, Critical Flicker Fusion; CIA, Clinical Impairment Assessment; CR, Caloric restriction; CS, Case Series; CT, Clinical Trial; DASS, Depression, Anxiety and Stress Scale; EDQ, Eating Disorders Questionnaire; ESS, Epworth Sleepness Scale; FAMS, Functional Assessment of Multiple Sclerosis; FS, Flourishing-Scale; FSS, Fatigue Severity Scale; GAD, Generalized Anxiety Disorder; GDS-15, Geriatric Depression Scale 15; HADS, Hospital Depression and Anxiety Scale; IF, Intermittent Fasting; IGF-1, Insulin Growth Factor I; IL-8, Interleukin 8; MCTQ, Munich Chronotype Questionnaire; MMP-9, Matrix Metalloproteinase 9; PANAS, Positive and Negative Affect Scale; PHQ-9, Patient Health Questionnaire–9; POMS, Profile of Mood State; PSQI, Pittsburgh Sleep Quality Index; PSS, Perception Stress Scale; RCT, Randomized Clinical Trial; RF, Ramadã Fasting; TEF, Time-Restricted Feeding; VAL, Voluntary Activation Level; VAS, Visual Analogue Scale; and WHO-5, World Health Organization-5 Well-Being Index.

## 4. Discussion

Preclinical studies have suggested a potential beneficial effect of IF on mood through actions on metabolic response, neurotransmission, the increased synthesis of neurotrophins, circadian rhythm, and the composition of the intestinal microbiota. However, investigations on the effects of IF in humans have enrolled mainly psychiatrically healthy volunteers and often assessed mood symptoms as secondary outcomes. Moreover, a great heterogeneity was observed regarding both the IF regimen studied and the observed effects on mood.

### 4.1. Studies Assessing IF in Animal Models of Mood Disorders

Eight rodent studies met the inclusion criteria of our systematic review [36,37,38,39,40,41,42,43]. Smith et al. evaluated, for 8 weeks, the effect of different IF protocols on insulin resistance in mice submitted to six different protocols: time-restricted feeding (TRF), alternate days fasting (ADF), Daniel fasting (DF), caloric restriction (CR), a high-fat diet (HFD), or an ad libitum diet. Except for HFD, all the other evaluated IF regimes (TRF, ADF, DF, and CR) were associated with an improved body composition, insulin sensitivity, and physical disposition (longer running time to exhaustion) [37]. Elesawy et al., observed that both healthy and diabetic rats submitted to a daily fast of 18 h for 3 months showed improvements in behavioral markers of anxiety and depression, in addition to showing increased neurotrophins BDNF and NT3 in the hippocampus. In addition, IF improved insulin resistance markers, decreased corticosterone levels, and increased serotonin, dopamine, and glutamate levels in the hippocampus, only in the group of rats with T2DM. These results can be explained by the fact that BDNF plays an important role in the regulation of energy consumption and expenditure, which can be highlighted by the fact that both BDNF and insulin receptors are coupled to the PI3-kinase-Akt and MAP kinase signaling pathways [37]. Interestingly, in this sense, Li et al. demonstrated that acute 9 h IF has a potential antidepressant effect in mice, in addition to potentiating the behavioral effect of the antidepressant imipramine in the animals. These effects would be mediated, at least in part, by HT2A/2C receptors (the administration of an agonist of these receptors reversed the behavioral effect of the intervention) and by the CREB-BDNF pathway in the hippocampus and prefrontal cortex [38]. Reinforcing previous findings on a potential beneficial effect of IF protocols on neuroplasticity markers, Dias et al. documented a better learning/spatial memory, higher expression of the Klotho gene, and higher neuroblast counts in the hippocampal dentate gyrus of mice subjected to ADF for 3 months compared to CR or ad libtum diet groups. In the same publication, the authors also described the results of an in vitro manipulation study with human hippocampal progenitor cells, in which the downregulation of the Klotho gene led to a reduction in neurogenesis, while the overexpression of the same gene produced an increase in neurogenesis [39]. The Klotho gene was originally identified as an aging suppressor gene, as animal studies have shown that its increased expression is associated with an increased life expectancy. In humans, the Klotho gene encodes the multifunctional protein α-Klotho, which participates in the regulation of phosphate, calcium, and vitamin D [59]. These findings are in line with other preclinical studies that found an increase in the expression of neurotrophins (such as BDNF) and the availability of neurotransmitters in the brain promoted by IF or moderate CR, which would be potentially implicated in increased synaptic plasticity and neurogenesis, improvements in depressive symptoms, and even the prevention of neuropsychiatric disorders [60,61]. In addition, the relationship between insulin resistance and neuroinflammation, mitochondrial dysregulation, neuroplasticity impairment, and depressive behaviors in animal models is now widely recognized [62], and the results observed in the present systematic review corroborate a potential beneficial effect of IF on mood by improving metabolism.

Strong evidence has suggested a bidirectional association between circadian rhythm and mental health, including the existence of animal studies documenting the association between circadian disruption (abnormalities in circadian rhythms) and depressive behaviors [63]. The circadian system in mammals has an approximate 24 h rhythm and regulates several physiological functions, including metabolism, feeding behavior, and the sleep–wake cycle. These clocks are regulated through feedback loops of transcription and the translation of clock genes, including Cry1/2, Per1/2, Bmal1, and Clock, generating an approximate rhythm of 24 h [64]. Mammals have a central clock, present in the suprachiasmatic nucleus of the hypothalamus (SCN) and synchronized by the light–dark cycle (night–day cycle), and peripheral clocks present in different organs. The SCN integrates and orchestrates the information coming from the various peripheral clocks, but these, in turn, can be synchronized by other stimuli, such as physical exercise, psychological stress, and food, with the timing and composition of the diet being important regulators of the peripheral clocks [65]. The animal studies found that evaluated circadian regulation pointed to the fact that TRF can be an effective tool for circadian synchronization, with potentially positive repercussions on metabolism and mood. Choi, Yamat, and Dallman observed that the circadian disruption induced by a colchicine injection into the SCN bilaterally produces a reduction in SLA (Spontaneous Locomotor Activity) and an increase in feeding behavior, water consumption, body weight, and insulin levels. On the other hand, the administration of TRF (2 h/day) was able to synchronize the circadian rhythm in the SCN, reducing food consumption and weight in rats even with circadian disruption induced by colchicine. The authors concluded that the ventromedial nuclei would act as an amplifier of the stimuli coming from the SCN and could represent a “food-adjustable oscillator” (circadian clock adjustable through the feeding period), while the SCN would integrate signals from other physiological oscillators [48].

Shim and Tanaka demonstrated that TRF promotes circadian synchronization regardless of exposure to light: rats with hereditary microphthalmia (blind) maintained the synchronization of their circadian phases when submitted to TRF (6 h/day), showing a peak of enzymatic activity (serotonin N-acetyltransferase, which catalyzes the production of melatonin from pineal serotonin) during the feeding period [41].

In order to evaluate the effect of TRF on mood changes promoted by external or occupational circadian disruption, Guerrero-Vargas et al. induced a shift–work situation (sleep deprivation during the resting phase) in rats in association with TRF during the active period. The authors observed that TRF was effective in preventing behavioral changes suggestive of anxiety and depression, in addition to reducing markers of neuroinflammation in the brain. This finding is particularly interesting in the context of findings from other studies on rodents, demonstrating that the disruption of circadian rhythms induced by a knock-down of the Bmal1 gene or forced desynchronization employing exposure to abnormal light–dark cycles increases depressive behaviors [66,67].

Finally, a single study considered the effect of IF on the composition of the intestinal microbiota. Soares et al. compared the effects of IF associated or not with aerobic exercises on anxious and depressive behaviors in rats. The authors observed that the animals submitted to IF in association with aerobic exercise showed a lower concentration of lactic-acid-producing bacteria in their feces and a tendency towards an anxiolytic effect, while the group of sedentary rats submitted to IF showed higher concentrations of the beneficial species Bifidobacterium and Enterococcus, associated with a tendency towards antidepressant effects. They concluded that IF and aerobic exercise, associated or not, are capable of positively modulating the MGBA [39]. This finding is in line with other animal studies on the impact of IF on the composition of the intestinal microbiota, which observed an increase in alpha-diversity, an improvement in the Firmicutes: Bacteroidetes ratio [68], and the restoration of diurnal variation in several families of bacteria involved in the absorption of nutrients, such as *Lactobacillus* and *Ruminococcacea*, which have been considered to be protective against the metabolic consequences of obesity [69].

### 4.2. Studies Assessing IF in Human

Daytime fasting with a night feeding period, in cases with RF, ends up having an anti-physiological impact on the individual’s circadian cycle. In our review, of the five included studies that evaluated RF, only one case series with healthy volunteers observed positive results in relation to mood [46]. Two case series noted a worsening in participants’ mood [44,45], and another case series even documented a reduction in blood BDNF levels during RF [47], despite the authors not reporting specific results for mood measures. Jahrami et al., in the only controlled clinical trial on patients with MDD identified in the present review, did not observe benefits of RF in terms of improving depressive symptoms [48]. With the understanding of the mechanisms related to circadian regulation obtained from the animal studies, we believe that the failure of most studies with RF published so far is due to the incongruity with circadian regulation [70]. During IF, feeding times become nocturnal, with two to three meals consumed after sunset [71]. This sudden and drastic change in the timing of food intake partially reverses the normal circadian pattern, leading to the disruption of biological clocks [70]. Several biological processes related to metabolism, digestion, and hormone secretion have a circadian rhythm [72,73]. Preclinical evidence has indicated that the alignment of feeding periods with the circadian rhythmicity of metabolic processes optimizes nutrition and benefits metabolic functions [74].

The physiological foundations that show the importance of circadian synchronization with the feeding window gives us indications that TRF may be the most effective fasting paradigm for mental health and mood control, since it promotes the alignment of the feeding interval to the appropriate circadian rhythm [74]. In line with this hypothesis, our review included the randomized, controlled clinical trial by Jamshed et al. which compared early TRF (8 a.m., between 7 a.m. and 3 p.m.) with an isocaloric control diet (12 h feeding window) for weight loss and body fat loss in obese individuals. Early TRF promoted a greater weight and body fat reduction, in addition to being associated with a significant improvement in fatigue/physical vigor and depressive symptoms [53].

Not only the relationship between TRF and circadian rhythm, but this revision also found that the time length is important as well. Interventions that extended beyond 12 weeks delivered better results in parameters that measured mood [53,54,55,56,57], which suggests that physiologic adaptations triggered by TRF need time to build up.

The studies that related eating habits to TDM converged in the sense that a diet rich in vegetables, whole grains, red fruits, nuts, and olive oil with a lower consumption of animal protein, such as in the Mediterranean diet, promote consistent benefits for depressive symptoms [8]. Based on these results, we believe that the quality of the diet, and not just the caloric value, can exert a great influence on mood, consisting of an important variable to be considered in clinical studies with IF. The lack of control over the quality of the diet during the IF can result in nutritional deficiencies and/or an increased intake of unhealthy foods, compromising the outcome of the intervention [75]. Furthermore, although studies consider IF to be a safe intervention, it is important to be careful when prescribing it, since individuals’ capacity for self-regulation is limited. This is an additional reason for the need for further studies with clinical populations of patients with mental disorders, as these present difficulties in adhering to the IF due to their cognitive and behavioral difficulties, even though they are good candidates for this type of intervention.

### 4.3. Limitations

Although the mechanisms identified in the animal studies are promising regarding the potential benefits of IF on mood in humans, different paradigms of IF may impact the mechanisms described above in different ways. This, together with the observed methodological limitations, may explain the great variability in the results found in the human studies included in this systematic review. The flourishing and recent scientific debate about the results of IF interventions for MDD, although promising, still lacks robust clinical studies, making it impossible to carry out a meta-analysis. In addition to the few studies, these present great methodological and intervention heterogeneity, preventing definitive conclusions about the effectiveness of different IF protocols in improving depressive symptoms. It is also relevant to note that only one investigation evaluated patients with MDD [44], while the others selected samples of psychiatrically healthy individuals or in whom the presence of mental disorders was not actively assessed. In these studies, mood was assessed as a secondary outcome of the IF intervention, and none of the clinical studies analyzed the nutritional quality of the diet.

## 5. Conclusions

Given the physiological effects of IF, it is plausible that it has indirect beneficial effects on mood through several pathways, such as through the control of metabolic parameters, body composition, the modulation of the intestinal microbiota, circadian synchronization, neurogenesis, and the increased availability of neurotransmitters. In our view, there are critical points that must be considered in the application of IF in clinical populations, such as a greater control of the quality of the diet, the practice of physical activity, the period of the feeding window, and the duration of fasting. The long-term effects of IF in humans also need to be further explored. We understand that the evidence found in this review, both in relation to the mechanisms better explored in animal studies and the results of interventions in humans, although heterogeneous or preliminary, is promising in relation to the potential benefits for the mood of IF protocols synchronized with the circadian rhythm. Thus, it is desirable that more studies—better designed, with larger samples and including patients with MDD—are carried out.
